# Cardiac safety of sofosbuvir-based direct-acting antivirals in hepatitis C patients with pre-existing heart disease^☆^

**DOI:** 10.1016/j.clinsp.2026.100908

**Published:** 2026-03-31

**Authors:** Amr Shaaban Hanafy, Mohamed Sorour Mohamed, Osama Attia, Ayman Fathy Elsayed Mohammed, Ahmad M. Hassaneen, Rania Naguib, Eslam Kamal Fahmy, Hany A. Elkattawy

**Affiliations:** a Internal Medicine Department, Gastroenterology and Hepatology Division, Zagazig University, Zagazig, Egypt; b Internal Medicine Department, Zagazig University, Zagazig, Egypt; c Clinical Pathology Department, Faculty of Medicine, Zagazig University, Zagazig, Egypt; d Department of Internal Medicine, College of Medicine, Princess Nourah bint Abdulrahman University, P.O. Box 84428, Riyadh, 11671, Saudi Arabia; e Department of Physiology, College of Medicine, Northern Border University (NBU), Arar, Saudi Arabia; f Department of Physiology, College of Medicine, Zagazig University, Zagazig, Egypt; g Department of Basic Medical Sciences, College of Medicine, Almaarefa University, Riyadh, Saudi Arabia; h Research Center, Deanship of Scientific Research and Post-Graduate Studies, AlMaarefa University, Riyadh, Saudi Arabia

**Keywords:** Sofosbuvir, Cardiac function, HCV, Outcome

## Abstract

**Background:**

Direct-Acting Antivirals (DAAs) are effective in 95% of patients, but concerns about potential cardiotoxicity and decreased Warfarin Sensitivity (WS) need further investigation. The study aimed to evaluate the safety and effectiveness of sofosbuvir-based therapy in patients with significant heart diseases, considering potential drug interactions and their impact on cardiac function.

**Methods:**

The study involved 100 patients with HCV genotype 4, including 40 with ischemic heart disease, 20 with prosthetic valve replacement, 20 with NYHA class I‒II heart failure, and 20 with systemic hypertension. All received sofosbuvir-based antiviral therapy and underwent routine laboratory tests, N-Terminal B-type Natriuretic Peptide (NT-Pro BNP), abdominal ultrasound, and echocardiography at baseline and three months post-treatment.

**Results:**

Ninety-six patients achieved SVR, resulting in improved cardiac ejection fraction (EF) from 53.2 ± 8.8% to 56.2 ± 6.9% (p = 0.000) and reduced left atrial volume (LAV) from 43.2 ± 4.2 to 41.7 ± 3.9 mm (p = 0.008). Liver stiffness also improved (p = 0.013), and no disabling symptoms were reported. MAPSE increased from 8.4 ± 2.4 mm to 9.5 ± 2.5 mm, and interventricular septum thickness (IVT) decreased from 12.5 ± 1.8 mm to 10.9 ± 1.3 mm (p = 0.001 and P = 0.000, respectively). The warfarin dose needed to be increased to optimize INR after SVR, but this adjustment did not affect WS (p = 0.16).

**Conclusion:**

Sofosbuvir-based antiviral therapy was well tolerated and demonstrated a reassuring cardiac safety profile in HCV patients with pre-existing heart disease, with no major arrhythmias or adverse cardiac events observed during or after therapy.

## Highlights

• Sofosbuvir improved cardiac function in chronic HCV patients with heart disease.
• Ejection fraction and left atrial volume improved, boosting heart performance.
• Liver stiffness significantly reduced after HCV eradication.
• Warfarin dosage was increased to optimize INR, maintaining stable anticoagulation.

## Introduction

Hepatitis C virus (HCV) is a major cause of chronic liver disease, affecting mortality and morbidity.^1^ It also affects the cardiovascular system, making it challenging to determine if the association between HCV infection and cardiac disorders is coincidental or causative. Studies suggest that HCV-related oxidative stress, inflammation, and cytopathogenic effects may influence cardiac cell function.^2^ Hepatitis C patients are more likely to develop coronary artery disease due to increased oxidative stress, a higher risk of diabetes, and viral replication in atherosclerotic plaques.^3^ Cardiomyopathies like acute myocarditis, fibrosis, and dilated cardiomyopathy are linked to HCV, with 6% of DCM patients testing positive.^4^ This association is linked to viral replication, TNF-alpha expression, and apoptosis. Hypertrophic cardiomyopathy is more common in HCV patients due to cryoglobulinemia and connexin-43 degradation.^5^^,^^6^ Portal hypertension and liver cirrhosis can cause cardiac dysfunction due to hemodynamic changes and neurohumoral abnormalities, increasing the risk of ventricular arrhythmia due to elevated cardiac output and autonomic dysfunction.^7^

Patients with HCV infection have a prolonged corrected QT interval, but those undergoing liver transplantation experience normalization. Patients with cirrhosis are more likely to develop supraventricular tachyarrhythmias.^8^ A study found that 17% of patients with HCV antibodies had a left ventricular EF below 50%, indicating a possible link between HCV and impaired cardiac EF.^9^

Direct-Acting Antivirals (DAAs) are efficacious in 95% of cases, although concerns regarding cardiotoxicity need additional research.^10^ Despite reports of Bradycardia, a meta-analysis identified no cardiovascular events in 1,625 individuals with sofosbuvir regimens.^11^^,^^12^ The claims of myocardial necrosis and affection of left ventricular ejection fraction with probable warfarin interaction were raised.^13^^,^^14^ This study aimed to evaluate the safety and effectiveness of sofosbuvir-based therapy in patients with significant cardiac diseases, considering potential drug interactions and their impact on cardiac function and morbidity.

## Subjects and methods

### Patient selection

The study included 100 patients (54 males and 46 females) with chronic active HCV genotype 4. All patients were classified as Child-Turcotte-Pugh class A, treatment-naive, but they presented with various significant cardiovascular disorders. Specifically, 40 patients had ischemic heart disease who were on warfarin due to [atrial fibrillation (n = 22), borderline EF (n = 12), previous DVT (n = 6)], 20 patients had undergone prosthetic valve replacement and were on warfarin therapy (including 12 with aortic valve replacements and 8 with mitral valve replacements), 20 patients had NYHA class I-II heart failure, and 20 patients had systemic hypertension with diastolic dysfunction and concentric left ventricular hypertrophy.

The study involved patients with active HCV diagnosed by HCV PCR and adequately controlled cardiac disease who received sofosbuvir-based antiviral therapy and were monitored in the outpatient clinic at Zagazig University Faculty of Human Medicine from February 2018 to July 2022. Patients showed a stable INR if they had two consecutive INR values within the therapeutic range of 2‒3 within 30-days of starting the HCV treatment regimen.

A non-treated control group was not included because withholding antiviral therapy from eligible HCV-infected patients would have been ethically unacceptable under current treatment standards. Therefore, a within-subject pre–post design was adopted, with all patients serving as their own controls.

Patients with liver diseases other than HCV, hepatocellular carcinoma, pregnancy, and a lack of effective contraception were excluded from the study. Other exclusions included those with a corrected QT interval of ≥ 450 ms, previous or family history of torsade de pointes, heart failure, recent myocardial infarction, severe uncontrolled hypertension, renal failure, untreated anemia, or those treated with amiodarone or propranolol.

### Laboratory analysis

Patients underwent laboratory evaluations to assess liver and renal function, blood counts, and coagulation profiles. Warfarin sensitivity was determined by dividing INR measurements by daily dose over seven days, at the end of treatment, and 12 weeks post-treatment.^15^

### Detection of N‐terminal B‐type natriuretic peptide

Blood samples were centrifuged and stored at -80°C. NT-pro BNP levels were measured using a double-antibody sandwich technique with electrochemiluminescence (Elecsys 2010, pro BNP, Roche Diagnostics). A serum concentration > 50 pg/ml was considered abnormal.

### FIB-4

It is calculated via the following equation: (age [years] × AST [U/L]) / (PLT [10^9^/L]) × (√ALT [U/L]). A FIB-4 score < 1.45 excludes advanced fibrosis in 90% of patients; however, a FIB-4 score > 3.25 has a specificity of 97% and a positive predictive value of 65% for advanced fibrosis.^16^

### Abdominal ultrasonography

Patients were examined for cirrhotic echo patterns, ascites, hepatocellular carcinoma, and abnormalities in the costophrenic angles indicative of pleural effusion and pericardial effusion.

### Liver stiffness measurement (LSM)

A physician conducted LSM using Fibroscan (Echosens, France); he was blinded to the patient's clinical data. LS values greater than 12.5 kPa indicated F4 or cirrhosis.^17^

### Echocardiographic examination

The operator, a member of a cardiology outpatient clinic, performed blind measurements at three time points: at baseline (time 0), at the end of treatment, and three months after the initiation of DAA treatment. A secondary blinded reader independently re‑analyzed a random subset (10%) of studies, yielding high interobserver agreement (ICC > 0.85), which supports the objectivity of these results. A comprehensive two-dimensional echocardiography evaluation was conducted using a Vivid 7 ultrasound scanner (General Electric Medical Health). The M-mode approach was used to measure posterior wall thickness and interventricular septum thickness (IVT), and to assess mitral annular plane systolic excursion (MAPSE), which is deemed abnormal if it is < 1.3 cm; values below 8 mm are associated with a depressed left ventricular EF. B-mode imaging was used to determine left ventricular end-systolic and end-diastolic diameters and volumes, as well as left atrial volume (LAV), all indexed to the body surface area.

The EF is calculated using the formula EDV - ESV / EDV. In diastolic dysfunction, the relationship between early and late filling was altered. The E/A ratio compares the peak velocity of blood flow during early diastole (E wave) to the peak velocity of blood flow during late diastole, caused by atrial contraction (A wave). Normal diastolic function for the 41-60-year age group is E > A: 1.28 ± 0.25. Diastolic dysfunction is classified into three grades: Grade 1, characterized by an E/A ratio of less than 0.8; Grade 2, which worsens diastolic dysfunction, leading to increased left atrial pressure and a larger E wave; and Grade 3, which often exceeds 2, potentially reaching 5 or higher.^18^

### Treatment protocol

Patients with portal hypertension and liver cirrhosis were prescribed sofosbuvir 400 mg, daclatasvir 60 mg, and ribavirin 600 mg daily based on the presence of a platelet count below 150,000/mcL, ultrasound findings consistent with cirrhosis, and liver stiffness greater than 12.5 kPa. Participants received a written and verbal explanation of potential cardiac risks, including arrhythmias, palpitations, and transient chest discomfort, before enrollment. A trained clinician ensured their understanding through a question-and-answer session, and informed consent was documented in accordance with ethical research protocols (Declaration of Helsinki). All patients decided to eradicate HCV, despite their cardiac conditions, through an informed consent form, explaining potential side effects.

### Follow-up

A clinical assessment was conducted to detect signs of hepatic decompensation, with laboratory investigations performed at weeks 2, 4, 8, and 12. At week 24, including CBC, creatinine levels, liver biochemical profile, and viral load, SVR is achieved when HCV RNA levels are below 15 IU/mL three months post-treatment termination. Virological nonresponse refers to persistently elevated HCV RNA levels above the detection limit; viral relapse occurs when these levels exceed the detection limit during follow-up after being previously negative. Treatment failure refers to adverse side effects that require discontinuation of treatment.

### Primary safety outcomes

The incidence and severity of adverse events, including discontinuation due to arrhythmia, worsening left ventricular systolic function, and acute coronary syndrome, were recorded. The effect of DAAs on left ventricular systolic function, pre-treatment WS, WS at the end of treatment, changes in INR, and warfarin dosage.

### Statistical analysis

The study used IBM SPSS version 25 for Windows (SPSS Inc., Chicago, USA). The Shapiro-Wilk test was used to determine whether the data were normally distributed. Continuous variables are presented as medians and interquartile ranges (IQRs) or means ± standard deviation (SD). For normally distributed data, an F-test was used, while paired t-tests were applied for parametric data. The Wilcoxon signed-rank test was used for nonparametric data. A p-value of less than 0.05 was considered statistically significant.

To control the family-wise error rate appropriately, multiple-comparison correction was applied within each clinical subgroup (IHD, prosthetic valve, CHF, hypertension/diastolic dysfunction). Each subgroup included 11 related comparisons (Warfarin dose, INR, WS, NT-proBNP, CRP, EF, MAPSE, LAV, IVT, E/A ratio, FibroScan). For transparency, the authors report raw p-values and use the Holm–Bonferroni step-down adjustment within each subgroup. A conservative per-family Bonferroni threshold (α family = 0.05/11 ≈ 0.004545) is used to indicate significance in the tables.

### Sample size consideration

This pragmatic cohort was based on the number of eligible HCV patients with pre-existing cardiac disease who attended our center during the study period. An a priori power calculation tied to a single prespecified endpoint was not feasible because the primary aim was exploratory to assess the safety and cardiac effects of DAAs therapy in a rare and heterogeneous population, a post-hoc analysis using the observed EF change (mean ± SD difference = 2.96 ± 6.32%) indicated an approximate standardized effect (Cohen’s d = 0.47) and > 99% power at α = 0.05. ^19^ Based on these parameters, a future prospective study seeking to detect a 5% EF difference would require approximately 13 patients for 80% power or 17 patients for 90% power using a paired design. These calculations are provided to guide future confirmatory research rather than to justify sample size retroactively.

## Results

The study involved 100 patients, with a mean age of 48.3 ± 8.6 years; of these, 31% were females. The patients showed mild increases in transaminase levels, with AST and ALT levels at 58 ± 15.6 and 58.7 ± 13.6 IU/L, respectively. The mean total serum bilirubin concentration was 1.41 ± 0.26 mg/dl. 97% of the participants had diabetes. The baseline FIB-4 score was 2.75 ± 1.73, and the baseline RNA was 944.8 ± 320.7 KIU/ml. Baseline CRP 45.2 ± 13.5 mg/l, LSM, as assessed by FibroScan, mean value 10.7 ± 3.8 kPa, 28 patients (28%) were classified as F0-F1, 28 patients (28%) as F2, 19 patients (19%) as F3, and 25 patients (25%) as F4. The mean baseline warfarin dose was 3.13 ± 1.94 mg, with a mean baseline INR of 1.9 ± 0.48 and a calculated WS of 0.44 ± 0.26.

Patients were classified into four categories depending on the aetiology of their cardiovascular illness. The IHD group was older, with higher serum transaminases, lower albumin levels, and lower platelet counts, which were most likely due to considerably higher viremia and HbA1c levels (p = 0.008). The prosthetic valve group exhibited increased total serum bilirubin and ammonia levels (p = 0.008). LSM levels were considerably higher in the IHD group (p = 0.008), which had a significantly higher frequency of F3 and F4 fibrosis phases (p < 0.000) (Table 1).

**Table 1:** Baseline demographic, laboratory, and elastography data of the studied subgroups.

Variable	IHD (n = 40)	Prosthetic valve (n = 20)	CHF (n = 20)	Hypertension + diastolic dysfunction (n = 20)	Adjusted P benforroni corrected	Eta Squared (η²)	95% CI
Sex (M/F)	27/13	12/8	14/6	16/4	1.0		
Age	53.4 ± 4.5	47.9 ± 4.8	51.3 ± 5.1	55.4 ± 6.7	0.008	0.201	0.060‒0.317
AST IU/L	69.4 ± 11.2	44.1 ± 12.5	59.5 ± 12.3	47.6 ± 10.7	0.008	0.465	0.305‒0.564
ALT IU/L	63.5 ± 11.1	50.8 ± 11.3	66.3 ± 13.3	49.3 ± 11.7	0.008	0.278	0.121‒0.394
Albumin gm/dL	3.8 ± 0.41	4.36 ± 0.6	4.14 ± 0.3	4.9 ± 0.26	0.008	0.335	0.172‒0.448
Total Bilirubin mg/dL	1.27 ± 0.2	1.66 ± 0.23	1.5 ± 0.26	1.34 ± 0.2	0.008	0.332	0.168‒0.444
HB gm/dL	11.9 ± 1.4	12 ± 1.42	11.9 ± 1.06	11.6 ± 1.07	1.0	0.012	0.002‒0.055
Platelet (10³/µL)	140 ± 48.9	152 ± 43.8	202.6 ± 17.7	210.9 ± 29.8	0.008	0.384	0.221‒0.493
FIB-4	2.93 ± 2.1	2.15 ± 0.98	1.9 ± 0.56	1.85 ± 0.6	0.008	0.317	0.155‒0.432
HbAIc %	7.65 ± 0.35	6.8 ± 0.74	7.08 ± 0.41	6.83 ± 0.91	0.008	0.291	0.133‒0.407
CRP (mg/L)	39.1 ± 13	60.1 ± 8.2	44.7 ± 10.3	43 ± 10.7	0.008	0.32	42.8‒47.2
Creatinine (mg/dL)	1.28 ±0.17	1.22 ± 0.09	1.03 ± 0.04	0.97 ± 0.07	0.008	0.549	0.402‒0.635
Serum Ammonia (µg/dL)	55.2 ± 7.24	74.1 ± 14.6	67 ± 7.7	54 ± 8.4	0.008	0.431	0.268‒0.534
PCR (RNA) (KIU/ML)	1108.9 ± 392.9	793.4 ± 159.4	861.7 ± 253.3	851.4 ± 180	0.008	0.181	0.046‒0.296
Warfarin dose	3.3 ± 0.52	5.8 ± 0.9	3.25 ± 0.64	‒	0.0003	0.911	0.874‒0.928
INR	2.13 ± 0.13	2.4 ± 0.24	1.5 ± 0.13	1.2 ± 0.13	0.008	0.897	0.855‒0.918
Warfarin sensitivity	0.66 ± 0.11	0.42 ± 0.08	0.48 ± 0.11	‒	0.0003	0.882	0.835‒0.905
LSM (kPa)	12.7 ± 4.4	9.1 ± 3.3	7.2 ± 2.4	8.4 ± 2.5	0.008	0.302	0.142‒0.417
Fibrosis stage, n (%)							
F0‒1	5 (12.5%)	6 (30%)	11 (55%)	6 (30%)			
F2	5 (12.5%)	7 (35%)	7 (35%)	9 (45%)	0.000		
F3	12 (30%)	3 (15%)	1 (5%)	3 (15%)			
F4	18 (45%)	4 (20%)	1 (5%)	2 (10%)			

### Baseline echocardiographic characteristics

The patients (n = 100) showed a fair baseline myocardial function, with a mean EF of 53 ± 8.8%, MAPSE of 8.3 ± 2.4, LAV of 43.2 ± 4.3 mm, IVT of 12.5 ± 1.8 mm, and an E/A ratio of 0.82 ± 0.17. The mean NT-pro BNP level was 89.9 ± 22.3 pg/ml. The study involved 67 patients with HCV who received treatment with sofosbuvir 400 mg and daclatasvir 60 mg daily for 3 months. Thirty-three patients with liver cirrhosis and portal hypertension were prescribed a triple therapy regimen with sofosbuvir 400 mg, daclatasvir 60 mg, and ribavirin 600 mg daily — a total of 96 out of 100 patients achieved SVR (96%).

**Fig. 1:**
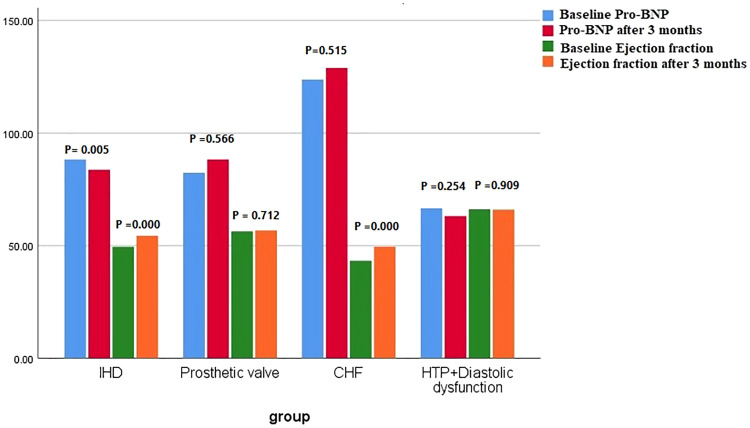
Comparison of the baseline values of NT-Pro -BNP and EF with the end-of-treatment values in the studied subgroups.

**Fig. 2:**
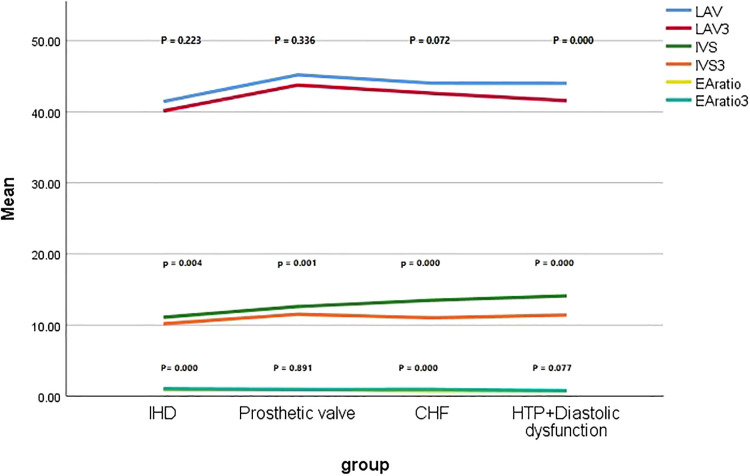
Comparison of baseline values of LAV, IVT and the EA ratio with end-of-treatment values across the studied subgroups.

**Fig. 3:**
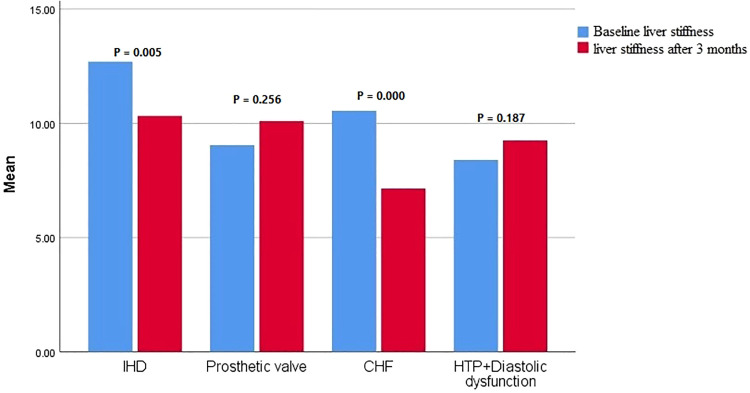
Comparison of baseline values of liver stiffness with end-of-treatment values in the studied subgroups.

In the IHD group, a slight increase in the warfarin dose was required (p = 0.0001), whereas the WS remained nearly stable (p = 0.835). A significant increase in EF and MAPSE (p = 0.000) (Fig. 1). While warfarin dosing changes (mean +0.4 mg were noted, there were no significant alterations in beta-blocker or ACE inhibitor usage or dosing during the study period. There was a non-significant decrease in LAV (p = 0.223) and a substantial reduction in IVT (p = 0.004), IVT Reduction is linked to better systolic function, reflects beneficial reverse remodelling which shows direct myocardial recovery, LAV reduction, primarily a marker of chronic diastolic dysfunction and atrial strain, occurs slowly and may lag behind improvements in myocardial wall thickness (Table 2, Fig. 2). LSM also improved at the end of treatment compared with the baseline value (from 12.7 ± 4.4 to 10.3 ± 1.6 kPa, p = 0.005) (Table 2, Fig. 3).

**Table 2:** Impact of DDAs treatment on liver stiffness and echocardiographic characteristics of the studied subgroups.

	IHD (n = 40)		p	Effect size (95% CI)
	Basal	3 months		
**Warfarin**	3.3 ± 0.52	3.7 ± 0.46	**0.0001(S)**	0.815 (0.17 ‒ 1.46)
**INR**	2.13 ± 0.13	2.38 ± 0.16	**0.000 (S)**	1.71 (0.99 ‒ 2.44)
**WS**	0.66 ± 0.11	0.65 ± 0.11	0.835 (**NS**)	-0.091 (-0.711, 0.529)
**NT-pro-BNP pg/mL**	88.3 ± 4.8	83.7 ± 8	**0.005 (NS)**^a^	-0.697 (-1.33, -0.059)
**CRP (mg/dL)**	39.1 ± 13	13.4 ± 5.7	**0.000 (S)**	25.6 (21.4 – 30)
**EF**	49.5 ± 4.3	54.4 ± 5.4	**0.000 (S)**	1.004 (0.346 ‒ 1.66)
**MAPSE**	6.64 ± 1.7	8.2 ± 1.4	**0.000 (S)**	1.002 (0.344 ‒ 1.66)
**LAV**	44 ± 2.9	42.6 ± 2.1	0.223 (**NS**)	-0.553 (-1.185, 0.079)
**IVT**	11.1 ± 1.4	10.2 ± 1.1	**0.004 (S)**	-0.715 (-1.35, -0.076)
**EA ratio**	0.89 ± 0.12	1.03 ± 0.14	**0.000 (S)**	1.07 (0.411 ‒ 1.74)
**Fibroscan(kPa)**	12.7 ± 4.4	10.3 ± 1.6	**0.005 (NS)**^a^	-0.725 (-1.36, -0.085)
	**Prosthetic valve (n** = **20)**		**p**	**Effect size (95% CI)**
	**Basal**	**3 months**		
**Warfarin**	5.8 ± 0.89	5.75 ± 0.85	0.86 (**NS**)	-0.057 (-0.934, -0.819)
**INR**	2.4 ± 0.24	2.44 ± 0.13	**0.52 (NS)**	0.207 (-0.672 ‒ 1.08)
**WS**	0.42 ± 0.08	0.43 ± 0.06	0.7 (**NS)**	0.141 (-0.736 ‒ 1.02)
**NT-pro-BNP pg/mL**	82.7 ± 7.23	88.3 ± 10	0.56 (**NS**)	0.642 (-0.257, 1.54)
**CRP (mg/dL)**	60.1 ± 8.2	15.8 ± 4.4	**0.000 (S)**	44.39 (39.7 ‒ 48.8)
**EF**	56.4 ± 4.03	56.8 ± 3.97	0.71 (**NS)**	0.1 (-0.777 ‒ 0.977)
**MAPSE**	9.14 ± 1.8	10.4 ± 3.46	0.196 (**NS**)	0.457 (-0.431 ‒ 1.35)
**LAV**	45.2 ± 4.9	43.8 ± 4.2	0.336 (**NS**)	-0.307 (-1.18, -0.575)
**IVT**	12.6 ± 1.4	11.5 ± 1.43	**0.001 (S)**	-0.777 (-1.68, -0.132)
**EA ratio**	0.93 ± 0.18	0.94 ± 0.14	0.891(**NS)**	0.062 (-0.815, -0.939)
**Fibroscan(kPa)**	9.1 ± 3.34	10.1 ± 1.7	0.256 (**NS**)	0.377 (-0.507, -1.26)
	**CHF (n** = **20)**		**p**	**Effect size (95% CI)**
	**Basal**	**3 months**		
**Warfarin**	3.3 ± 0.64	3.9 ± 0.37	**0.002 (S)**	1.148 (0.202 ‒ 2.09)
**INR**	1.5 ± 0.13	2.1 ± 0.4	**0.000 (S)**	2.02 (0.941 ‒ 3.09)
**WS**	0.48 ± 0.11	0.55 ± 0.13	0.109 (**NS**)	0.581 (-0.314, 1.48)
**NT-pro-BNP pg/mL**	124 ± 23.6	129 ± 30	0.515 (**NS**)	0.185 (-0.693 ‒ 1.06)
**CRP (mg/dL)**	44.7 ± 10.3	14..8 ± 4.9	**0.000 (S)**	30 (23.7 ‒ 36)
**EF**	43.3 ± 2.32	49.5 ± 3.1	**0.000 (S)**	2.26 (1.14 ‒ 3.38)
**MAPSE**	7.9 ± 0.9	9.14 ± 1.8	**0.007(NS)**^a^	0.871 (-0.046, 1.78)
**LAV**	44 ± 2.9	42.6 ± 2.1	0.072 (**NS)**	-0.553 (-1.446, 0.34)
**IVT**	13.5 ± 1.1	11 ± 1.45	**0.000 (S)**	-1.94 (-3.0, -0.879)
**EA ratio**	0.69 ± 0.11	0.95 ± 0.18	**0.000 (S)**	1.74 (0.713 ‒ 2.77)
**Fibroscan(kPa)**	10.6 ± 1.4	7.2 ± 2.4	**0.000 (S)**	-1.73 (-2.75, -0.703)
	**Hypertension with severe diastolic dysfunction (n** = **20)**		**p**	**Effect size (95%CI)**
	**Basal**	**3 months**		
**Warfarin**	‒	‒	‒	
**INR**	1.17±0.13	1.4±0.27	**0.001(S)**	1.085 (0.147, 2.02)
**WS**	‒	‒	‒	
**NT-pro-BNP pg/mL**	66.6 ± 8.8	63 ± 7.2	0.254(**NS**)	-0.448(-1.335, 0.44)
**CRP (mg/dL)**	43 ± 10.7	14.8 ± 6.9	**0.000 (S)**	28.4 (21.6 ‒ 35.2)
**EF**	66.2 ± 4.6	66 ± 2.7	0.909 (**NS**)	-0.053 (-0.93, -0.824)
**MAPSE**	11.4 ± 1.7	11 ± 2,7	0.606(**NS**)	-0.177 (-1.056, 0.701)
**LAV**	44 ± 1.8	41.6 ± 2	**0.000 (S)**	-1.26 (-2.22, -0.302)
**IVT**	14.1 ± 1.1	11.4 ± 0.7	**0.000 (S)**	-2.92 (-4.19, -1.67)
**EA ratio**	0.7 ± 0.16	0.77 ± 0.1	0.077(**NS**)	0.525 (-0.367, 1.42)
**Fibroscan(kPa)**	8.4 ± 2.5	9.3 ± 1.6	0.187(**NS**)	0.429 (-0.458, 1.32)

*CRP, C reactive protein; EF, Ejection Fraction; HCV, Hepatitis C Virus; INR, International Normalized Ratio; IVT, Interventricular Septum; LAV, Left Atrial Volume; LSM, Liver Stiffness Measurement; MAPSE, Mitral Annular Plane Systolic Excursion; NT-Pro BNP, N‐Terminal B‐type Natriuretic Peptide; WS, Warfarin Sensitivity; S, Significant if p < 0.00143 (Holm-Bonferroni correction); NS, Non-significant if p > 0.00143 (Holm-Bonferroni correction).

a Shows which parameters became non-significant after subgroup-wise Holm correction.

With 11 primary comparisons, the authors used the Holm–Bonferroni step-down adjustment within each subgroup, employing a conservative per-family Bonferroni threshold (αfamily = 0.05/11 ≈ 0.004545). Therefore, only results with p < 0.004545 remain statistically significant. Consequently, all considerable variables hold significance, except for Fibroscan reading and NT-proBNP, which decreased from 88.3 ± 4.8 to 83.7 ± 8 pg/mL (raw *p* = 0.005). This absolute change (∼4.6 pg/mL) is below the expected intra-assay and biological variability for NT-proBNP and did not remain significant after Holm–Bonferroni correction within the IHD subgroup (adjusted threshold P ≈ 0.004545); therefore, the NT-proBNP change should be interpreted as a nominal biochemical trend rather than a clinically meaningful effect (Table 2). None of the participants reported disabling symptoms such as dyspnea, systemic or pulmonary congestion, or cardiac arrhythmia.

In patients who underwent prosthetic valve replacement, DAAs did not significantly affect the warfarin dosage (p = 0.86), INR (p = 0.52), or warfarin sensitivity (p = 0.7). There was a non-significant improvement in MAPSE, EF, and LAV (Fig. 1, Fig. 2), along with a significant reduction in IVT (P = 0.001), which held substantial after the Holm-Bonferroni method (Fig. 2). However, there was no effect on LSM by FibroScan (p = 0.256) (Table 2, Fig. 3).

DAAs in patients with CHF significantly influenced the warfarin dosage, increasing it from 3.3 ± 0.64 mg to 3.9 ± 0.37 mg (p = 0.002). Additionally, there were significant improvements in EF (p = 0.000), non-significant in MAPSE (p = 0.007, after subgroup-wise Holm correction) (Table 2, Fig. 1), EA ratio and IVT (p = 0.000) (Fig. 2) with a significant reduction in LSM (p = 0.000) (Table 2, Fig. 3).

Patients with hypertension and diastolic dysfunction demonstrated significant improvements in LAV (p = 0.000) and IVT (p = 0.000) at the end of treatment, which remained significant after correction (Table 2, Fig. 2). Notably, these improvements occurred without any considerable side effects.

CRP, as a marker of systemic inflammation in HCV patients, was evaluated and showed a highly significant improvement in all patient subgroups at the end of treatment. This improvement remained significant after Holm–Bonferroni correction (Table 2). While useful as a broad inflammatory indicator, CRP is non-specific and may be influenced by hepatic improvement.

Following DAA therapy, SVR was associated with significant adjustments in warfarin dosage (p = 0.004), which were required to maintain therapeutic INR levels (p = 0.000) (Table 3), though WS remained stable (p = 0.16). This stability confirms the absence of any pharmacokinetic or pharmacodynamic interference between DAAs and warfarin.

**Table 3:** Impact of achieving SVR on laboratory, echocardiographic and liver stiffness data of the studied patients.

	Baseline (n = 96)	SVR (n = 96)	95% CI	p
**Warfarin (mg)**	3.13 ± 1.98	3.4 ± 1.98	-0.24 (-0.4 ‒ -0.08)	0.004
**INR**	1.85 ± 0.49	2.12 ± 0.46	-0.27 (-0.34 ‒ -0.2)	0.000
**WS**	0.43 ± 0.261	0.45 ± 0.27	-0.019 (-0.05 ‒ 0.01)	0.16
**NT-ProBNP pg/mL**	89.96 ± 22.7	89.99 ± 26.8	-0.035 (-3.8 – 3.77)	0.985
**EF (%)**	53.2 ± 8.8	56.2 ± 6.9	-2.96 (-4.2 – -1.68)	0.000
**MAPSE (mm)**	8.4 ± 2.4	9.5 ± 2.5	-1.03(-1.6 ‒ -0.4)	0.001
**LAV (mm)**	43.2 ± 4.2	41.7 ± 3.9	1.5 (0.4 – 2.6)	0.008
**IVT (mm)**	12.5 ± 1.8	10.9 ±1.3	1.67 (1.33 – 2.02)	0.000
**EA ratio**	0.82 ± 0.17	0.93 ±0.17	-0.12 (-0.16 – -0.07)	
**Fibroscan (kPa)**	10.6 ± 3.8	9.4 ± 2.2	1.15 (0.24 – 2.05)	
**CRP (mg/L)**	45.3 ± 13.2	14.4 ± 5.6	30.8(28 – 33.7)	

*CRP, C Reactive Protein; EF, Ejection Fraction; INR, International Normalized Ratio; IVT, Interventricular Septum; LAV, Left Atrial Volume; LSM, Liver Stiffness Measurement; MAPSE, Mitral Annular Plane Systolic Excursion; NT-Pro BNP, N‐terminal B‐type Natriuretic Peptide; WS, Warfarin Sensitivity.

Raw data was published at: https://zenodo.org/records/15835098

Left ventricular EF increased significantly (p = 0.000), indicating preserved or slightly enhanced systolic function in a population predisposed to functional decline. Although this increment remained within the normal range and below the threshold for prognostic benefit, it supports the overall cardiac safety of therapy (Table 3).

The E/A ratio showed a significant increase (p = 0.000), reflecting a trend toward normalization of early diastolic filling patterns and improved left ventricular compliance. These findings, along with stable volume management and unchanged diuretic regimens throughout treatment, suggest that hemodynamic stability was maintained under optimized heart failure care.

SVR was also associated with a significant reduction in left atrial volume (LAV: 43.2 ± 4.2 → 41.7 ± 3.9 mL, p = 0.008). Although modest, this reduction is given the typically slow nature of atrial remodelling and may indicate lower filling pressures and better diastolic function. IVT decreased significantly (p = 0.000), consistent with early reverse remodeling and improved wall compliance, suggesting partial regression of concentric hypertrophy and more favourable hemodynamic load.

MAPSE increased significantly (p = 0.001), reflecting enhanced longitudinal systolic motion. Because MAPSE values below 8 mm usually denote impaired contractility, this improvement indicates recovery toward the lower-normal range, consistent with better myocardial deformation and diastolic–systolic coupling.

A significant decline in liver stiffness, as measured by FibroScan (p = 0.013), suggested regression of hepatic inflammation and early improvement in fibrosis, which may have contributed indirectly to more balanced cardio-hepatic interactions and attenuation of hyperdynamic circulation. In parallel, CRP levels decreased markedly (p = 0.000), reflecting attenuation of systemic inflammation and indirectly supporting improved vascular and myocardial homeostasis.

General Linear Model (GLM) procedures were performed. These allow for the comparison of group means (changes in IVT baseline, after 3 months, LAV baseline, and after 3 months) while controlling for changes in warfarin dose. These models demonstrated that the association between DAA therapy and SVR, as well as improvements in IVS thickness and LAV, remains statistically significant (IVS, IVS3, LAV, LAV3) (P = 0.000, 0.008, 0.009, 0.028, respectively).

## Discussion

Recent advancements in HCV treatment, particularly sofosbuvir-based DAA therapy, have been effective, better tolerated, and safer than earlier interferon-based regimens. However, there is limited literature on the potential adverse effects of these drugs on cardiac function in patients with clinically significant cardiac diseases. The current study investigated the safety of DAAs in patients with HCV and other cardiovascular disorders, finding a SVR rate of 96% and no significant adverse effects on cardiac function in this subgroup of HCV patients.

The study involved 100 patients with significant cardiac conditions, including IHD, prosthetic valve replacement, CHF, and systemic hypertension with diastolic dysfunction. After three months of sofosbuvir-based therapy, all patients completed the treatment without significant changes in quality of life or heart electrophysiologic or contractile functions, and their cardiac EF improved from 53.2 ± 8.8 to 56.2 ± 6.9% (p = 0.000).

A study found that sofosbuvir-based therapy for HCV treatment in thalassemia patients was safe, and no significant change in EF was observed after treatment.^20^ Another study found no change in EF in patients with chronic HCV infection who did not have cardiac risk factors and were using NS5A inhibitors.^21^

This study found no significant deterioration in LAV among high-risk patients. Still, a substantial decrease in LAV was observed in patients with hypertension and diastolic dysfunction from 44 ± 1.8 before therapy to 41.6 ± 2 after treatment. This improvement may reflect a better prognosis for those who received SOF-based therapy and achieved sustained SVR, as LAV serves as an indicator of diastolic burden and predicts clinical outcomes and mortality.^22^ Poller et al. found that eradicating HCV through DAAs therapy improved cardiac function in a patient with advanced heart failure listed for cardiac transplantation, reducing the NYHA functional class from II-IV to class II.^23^ Recent studies have shown no significant changes in EF, global longitudinal strain, and wall motion abnormalities after sofosbuvir-daclatasvir combination therapy, while improving metabolic parameters, mainly insulin resistance.^24^

Although NT-proBNP showed a small nominal decrease in the IHD group, the absolute change (∼4.6 pg/mL) is below the limits of analytic and biological variability and lost statistical significance after appropriate within-subgroup multiple-comparison correction. The authors therefore regard this finding as clinically trivial and hypothesis-generating. Any suggestion of improved myocardial strain should rest primarily on the constellation of the concordant conclusions (echocardiographic measures and CRP) rather than this isolated biochemical result.

In the current study, although the EF increase was modest in IHD and CHF subgroups, (49.5-> 54.4% & 43.3→ 49.5%, respectively), (p = 0.000), and in patients who achieved SVR (n = 96), EF improved from 53.2 ± 8.8 to 56.2 ± 6.9 (p = 0.000), although the absolute increase in EF (≈3%) is below the conventional threshold for clinical significance, the parallel improvement in MAPSE, IVT, NT-proBNP, and CRP suggests a consistent biological trend rather than random fluctuation. These subtle improvements may represent early myocardial recovery following HCV eradication in patients with chronic cardiac disease, suggesting that chronic active HCV infection may contribute to myocardial dysfunction.^25^^,^^26^

Among the enrolled patients, none developed arrhythmia during treatment or after achieving SVR, in line with recent studies, which indicated that sofosbuvir-based therapy does not increase the risk of bradyarrhythmia.^27^^,^^28^ Arrhythmia occurred with DAA therapy, possibly due to drug-drug interactions with amiodarone. It is also important to note that these studies involved ledipasvir, whereas the studied patients were treated with daclatasvir.^29^^,^^30^

The study found a significant improvement in cardiac parameters like MAPSE, mainly in the IHD group, and cardiac EF in the IHD and CHF subgroups, while decreasing IVT (CHF, prosthetic valves, Hypertension &diastolic dysfunction subgroups).

Warfarin dose requirements showed a modest, statistically significant increase in some subgroups, while INR values and warfarin sensitivity remained stable. No bleeding or thrombotic events occurred, and all patients maintained therapeutic anticoagulation through routine INR monitoring. These findings do not represent a novel mechanistic observation but rather confirm standard clinical practice. Importantly, they provide reassuring evidence of the absence of clinically relevant pharmacokinetic or pharmacodynamic interactions between sofosbuvir-based direct-acting antivirals and warfarin. This is particularly relevant because patients on chronic anticoagulation are frequently excluded from DAA clinical trials. The present results therefore support the clinical safety and feasibility of administering DAAs in cardiac patients requiring long-term warfarin therapy, provided that INR is monitored according to standard protocols.

The study's limitation is that it was conducted in a single centre; a larger number of patients should have been included, but this was due to the rarity of this subset of HCV patients eligible for treatment. Subgroup sizes (∼n = 20) are small. This sample size likely reduces the authors’ ability to detect moderate changes and may contribute to the non-significant results observed (e.g., LAV change in IHD, p = 0.223). This study lacked an a priori sample-size calculation based on a prespecified primary endpoint due to its pragmatic, feasibility-based design. Although post-hoc analyses indicate high statistical power for the observed EF change, these results should be interpreted as exploratory. Future confirmatory trials should define a single primary outcome and perform formal a priori sample-size estimation to establish clinical significance. The absence of a non-treated control group represents a limitation, which was ethically precluded since withholding antiviral therapy from eligible HCV-infected patients would contravene current treatment standards. Nevertheless, a within-subject pre–post design allowed each patient to serve as their own control, and all participants were maintained on stable cardiac medications during follow-up. In addition, multivariable generalized linear modelling was applied to adjust for potential confounders, thereby reducing the likelihood that observed improvements were due solely to background cardiac management. Additionally, the observed changes are small and should be considered hypothesis-generating rather than confirmatory; therefore, confirmatory studies with longer follow-up are required to determine whether they translate into meaningful clinical outcomes.

This was the first study to investigate the impact of DAAs on specific cardiac parameters, including MAPSE, EF, IVT, and LAV. The most consistent and clinically meaningful finding of this study was the excellent cardiac safety profile of sofosbuvir-based therapy in a cohort of patients with significant cardiovascular comorbidities. Despite theoretical concerns regarding cardiotoxicity and potential drug interactions, no arrhythmias, ischemic events, or deterioration in ventricular function were observed. These results provide real-world reassurance regarding the safety of DAA therapy in a population typically underrepresented in pivotal clinical trials. Future studies are needed to evaluate the safety and cardiac effects of DAAs in patients with advanced liver disease (Child–Pugh B/C) or decompensated heart failure (NYHA III–IV), to determine whether the observed cardiac improvements extend to these higher-risk groups.

## Conclusions

Sofosbuvir-based DAA therapy appeared safe and was associated with stable cardiac function and modest favourable trends in patients with chronic HCV infection and pre-existing heart disease. These changes are likely consistent with the effects of stable cardiac care and reduced systemic inflammation rather than a direct myocardial action of DAAs. Because a causal relationship between HCV eradication and cardiac improvement cannot be established from this observational study, the findings should be interpreted as exploratory and hypothesis-generating. Larger, controlled, and mechanistically focused studies are warranted to confirm and elucidate these associations.

## Patient consent for publication

Patients provided their consent for their data to be used for publication and research purposes, without revealing their identities.

## Ethics approval

The study methods were approved by the Research Ethics Board of the Zagazig Faculty of Medicine, Egypt (IRB: 863/26/24). The research was conducted ethically in accordance with the World Medical Association Declaration of Helsinki and its later amendments.

## Authors’ contributions

Conceptualization: AS Hanafy, HA Elkattawy, EK Fahmy; Data curation: all authors, Formal analysis: MS Mohamed, O Attia, AF Elsayed; Methodology: AS Hanafy, AM Hassaneen. Writing original draft: R Naguib; Writing review & editing: all authors. Approval of final manuscript: all authors.

## Funding

The study was funded by 10.13039/501100004242Princess Nourah bint Abdulrahman University Researchers Supporting Project number (PNURSP2026R774), Princess Nourah bint Abdulrahman University, Riyadh, Saudi Arabia.

## Data availability statement

The datasets generated and/or analyzed during the current study are available from the corresponding author upon reasonable request.

## Declaration of competing interest

The authors declare that they have no conflicts of interest. The authors declare that the funders had no role in the study design, data collection, analysis, or interpretation; manuscript drafting; or the decision to publish.
